# Golden jackals as hosts of zoonotic parasites: preliminary findings from southern Poland

**DOI:** 10.2478/jvetres-2026-0018

**Published:** 2026-03-31

**Authors:** Katarzyna Bojarska, Katarzyna Kondratek, Aleksandra Leszczyńska, Karolina Sionek, Jennifer Hatlauf, Jacek Karamon, Małgorzata Samorek-Pieróg, Henryk Okarma, Stanisław Śnieżko, Anna Didkowska, Blanka Orłowska, Anna M. Pyziel

**Affiliations:** Institute of Nature Conservation, Polish Academy of Sciences, 31-120, Kraków, Poland; The Scientific Society of Veterinary Medicine Students, Department of Food Hygiene and Public Health Protection, Institute of Veterinary Medicine, Warsaw University of Life Sciences, 02-776, Warsaw, Poland; Institute of Wildlife Biology and Game Management, BOKU University Vienna, 1180, Vienna, Austria; National Veterinary Research Institute, 24-100, Puławy, Poland; Department of Food Hygiene and Public Health Protection, Institute of Veterinary Medicine, Warsaw University of Life Sciences, 02-776, Warsaw, Poland; Department of Public Health Protection and Animal Welfare, Faculty of Biological and Veterinary Sciences, Institute of Veterinary Medicine, Nicolaus Copernicus University in Toruń, 87-100, Toruń, Poland

**Keywords:** *Canis aureus*, range expansion, Poland, potential helminthic zoonosis, syntopic detection

## Abstract

**Introduction:**

The golden jackal, Canis aureus, has been rapidly expanding its range across Europe, raising concerns regarding its impact on the health of wildlife, domestic animals and humans. The goal of this study was to examine the parasitological fauna of golden jackals that had recently colonised southern Poland.

**Material and Methods:**

The direct flotation method with centrifugation was used to search for parasite eggs, and a complex PCR and qPCR were run to detect the presence of tapeworms in nine faecal samples found by dogs and confirmed genetically, and in two samples taken from hunted individuals. The intestine contents of the hunted individuals were also examined using the sedimentation and counting technique. Camera traps were set to assess if the activity of golden jackals overlapped spatially with that of domestic animals.

**Results:**

Nine out of eleven faecal samples contained eggs of the Toxocara and Trichuris genera and the Capillariidae and Ancylostomatidae families. Mesocestoides litteratus DNA was detected in three faecal samples and Taenia serialis DNA in one sample collected from a hunted golden jackal. The sites frequented by domestic cats and dogs were also visited by golden jackals.

**Conclusion:**

Although golden jackals may be suspected of contributing to the transfer of some parasites to humans through domestic animals, the findings do not indicate that golden jackals represent a significant current or emerging threat to the health of wildlife or humans in southern Poland.

## Introduction

Fast range expansions and the subsequent appearance of species in new areas may have deep ecological consequences that affect whole ecosystems (8, 21). They also pose challenges for nature conservation and management, especially for species associated with human-wildlife conflicts (29, 38) or the potential spread of zoonoses (10, 37, 47, 49). The recent expansion of the golden jackal (*Canis aureus*) is an example of such a phenomenon. Only in the past decade, the golden jackal has expanded its range to over 12 new European countries, and it is currently considered the fastest-spreading mammalian species in Europe (19). The rapid expansion of the golden jackal is attributed to global change, including climate warming and land use changes (2, 40, 44). The speed of the species’ expansion was not predicted by scientists or policy decision makers, and is currently igniting discussions regarding its potential impact on biodiversity, the economy and pathogenic agents (18).

Canids can play the role of definitive hosts for many intestinal parasites, and they serve as the source of some serious parasitic diseases with a zoonotic character, including nematodes of the *Toxocara, Ancylostoma, Uncinaria, Trichuris* and *Strongyloides* genera; trematodes of the *Alaria* genus; tapeworms of the *Dipylidium* and *Echinococcus* genera; and other human soil-transmitted helminthiases (17, 45). Wild canids, such as the red fox (*Vulpes vulpes*), the wolf (*Canis lupus*), and the raccoon dog (*Nyctereutes procyonoides*), are hosts of high epidemiological significance, as they can spread the eggs, larvae and other dispersive forms of zoonotic parasites into the environment, where they can represent a risk factor for domestic animals and humans (1, 6, 24). Lately, because of the golden jackal’s rapid expansion and long-distance dispersal (4, 39), it has been receiving increased attention regarding its potential to spread zoonoses.

Recent findings have indeed shown that the expansion of golden jackals in Europe can worsen the spread of several zoonotic diseases (27) and thus influence the characteristics of their endemicity in host species, including humans (3). The golden jackal’s generalist feeding habits and broad dietary spectrum expose it to potential interactions with numerous parasitic organisms, rendering it a potential vector for these pathogens (32). Prior investigations (17) reported that golden jackals act as hosts for helminth parasites capable of infecting domestic dogs and cats, such as *Toxocara* or *Uncinaria* spp., which can subsequently be transmitted to humans. Moreover, these canids can be definitive hosts of *Echinococcus* spp., parasites extremely dangerous to humans, which has already been confirmed in Europe (3, 15, 36, 46). To date, no parasitological research has been conducted on golden jackals in Poland, which makes it imperative to investigate the presence of gastrointestinal parasites in this species.

The golden jackal’s presence in Poland was first documented in 2015 (30). In 2017, the Ministry of the Environment listed it as a game species, allowing either seasonal or (in some areas) year-round hunting. However, basic knowledge about the species’ ecology, behaviour and pathogens in recently colonised areas is still insufficient. Up to 2022, only 19 observations of golden jackals (either dead individuals or high-quality photographs) scattered all over the country had been documented (23), and the reproduction of this species has been reported only once (31). In 2021, we obtained information from local hunters about regular observations of a group of golden jackals in the area of the Nysa Reservoir (southern Poland), indicating a second reproduction event of this species in the country. The aim of this study was to examine parasitological fauna in golden jackal faeces and investigate camera trap material to study the potential role of the golden jackal as a vector of zoonotic diseases.

## Material and Methods

### Study area and sampling

**Fig. 1.**
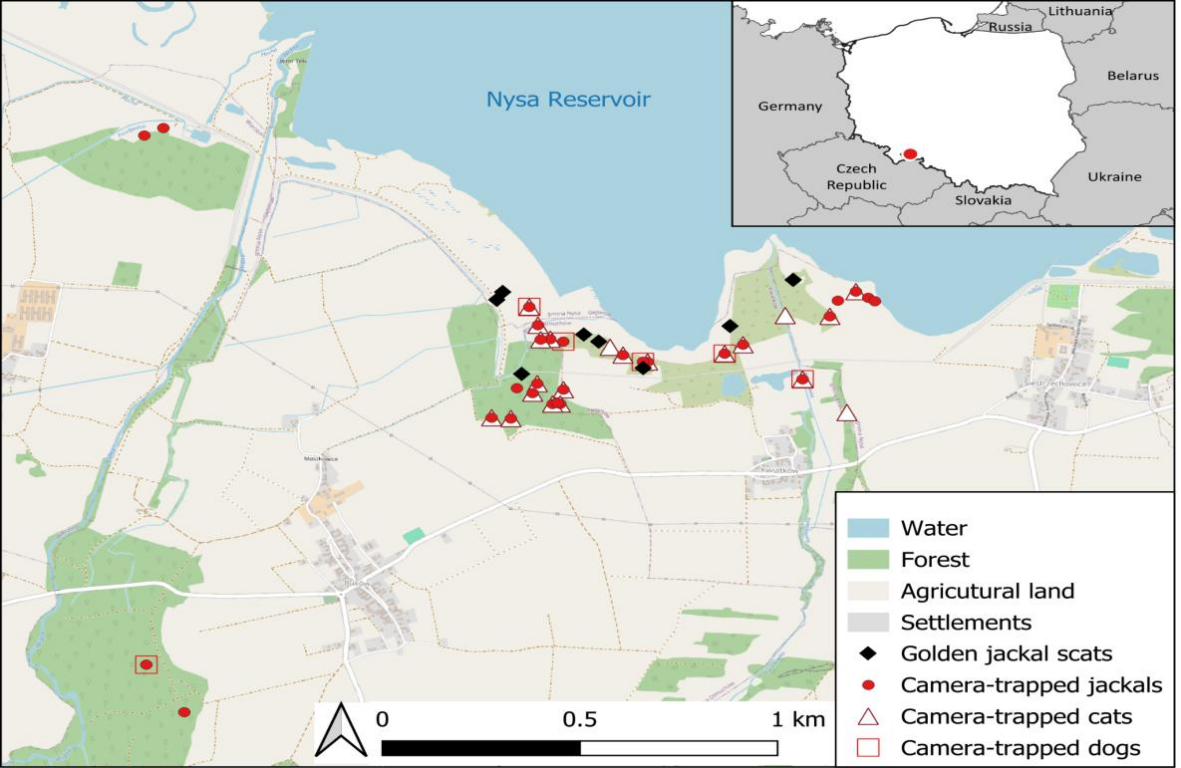
Locations of golden jackal scat samples used for parasitological analyses and locations of camera traps where golden jackals, domestic cats and dogs were recorded in southern Poland, 2022

The present study was conducted in southern Poland, south of the Nysa Reservoir, in Otmuchów municipality (17.23°E, 50.44°N) (Fig. 1). The study area encompassed 15 km^2^, with habitats ranging from seasonally flooded shrubby marshes to patches of deciduous woodlands and open agricultural areas. Apart from the golden jackal, the mesocarnivore community in the area also consists of the red fox (*Vulpes vulpes*), European badger (*Meles meles*), Eurasian otter (*Lutra lutra*) and raccoon dog (*Nyctereutes procyonoides*).

In February 2022, preliminary bioacoustic stimulation was undertaken at eight preselected points and the presence of at least one golden jackal family group of at least three individuals was confirmed. During the following three days, three specially trained scat-detection dogs and their handlers searched for golden jackal faeces along 154 km of transects, as reported by Hatlauf *et al*. (20). All scats found by the dogs were divided into subsamples for genetic and parasitological analyses. The faecal samples for parasitic analyses were placed into two separate 30-mL labelled plastic tubes and stored in a portable cooler during transport to the laboratory. Additionally, two faecal samples were taken post mortem from hunted females, one from a jackal shot in autumn 2023 which weighed 10.9 kg and the other from a carcass in February 2024 weighing 10.0 kg.

### Molecular identification of host species from faecal samples

Humans being unable to reliably distinguish by visual inspection between golden jackal faeces and those from other similarly sized canid species, especially those of the red fox (20), genetic identification of the species was made based on mitochondrial DNA (primers L15995, WdloopH and WdloopL) (7). The confirmed golden jackal samples were further analysed to identify the individuals based on 13 autosomal microsatellite loci and two sex markers: CPH5 (14); FH2001, FH2010, FH2017, FH2054, FH2087, FH2088, FH2097, FH2137, FH2140 and FH2161 (13); vWF (43); PEZ17 (35); and DBX6 and DBY7 (42). The laboratory procedures of DNA extraction and genotyping were performed at the laboratory of Senckenberg Gesellschaft für Naturforschung, Germany, and followed the protocols described in Hatlauf *et al*. (20).

### Parasitological examination of faecal samples

A modified version of the Willis method was used, which involved the direct flotation method with centrifugation (16). The subsamples for parasite analyses (0.24 g–4.23 g) were passed through a sieve while suspended in 10 mL of sucrose solution with a specific gravity of 1.27. The resulting suspension was transferred into 10 mL centrifuge tubes and centrifuged for 2 min at 2,000 rpm. Next, the samples were placed vertically in test tube racks and an additional sucrose solution was added to each tube, creating a noticeable bulging meniscus. Glass coverslips were placed on the top of the menisci and allowed to rest for 20 min. The coverslips were subsequently laid onto glass slides, and the samples were examined under a LAB40 (Opta-Tech, Warsaw, Poland) light microscope at 100× and 400× magnifications. Microphotographs of parasitic eggs were taken directly from the microscope using the OPTA View-15 2019 (Opta-Tech) imaging software package. The eggs of parasites were determined to the genus or family level. The number of eggs found on the slides was converted to content per gramme.

### Parasitological examination of the intestines

The intestines of the hunted individuals were examined using the sedimentation and counting technique (SCT) (22). Faeces were additionally collected from the rectum and examined using the flotation method described above.

### Molecular screening for tapeworm presence

The faecal samples’ content of DNA was extracted using a QIAamp Fast DNA Stool Mini Kit (Qiagen, Hilden, Germany) according to the manufacturer’s protocol for larger stool volumes. Next, the isolates were examined with the following methods: a qPCR for the detection of *E. multilocularis* (28); a qPCR for *E. granulosus s.l*. (34); and a multiplex PCR for *E. multilocularis, E. granulosus s.l*., *Taenia* spp. and some other tapeworms, including *Mesocestoides* sp. (48). Positive products obtained in PCR were sequenced *via* Sanger dideoxy sequencing at a commercial company (Genomed, Warsaw, Poland) and the received sequences were compared with GenBank databases.

### Camera trapping

Camera traps (Bolyguard SG520, Boly Media, Shenzhen, China) were set to monitor the co-occurrence of golden jackals and domestic animals. From April 2022 to June 2023, 33 sites were monitored for various periods (1–14 months), during a total of 6,299 camera-trap days. The cameras were set for continuous activity and 30-s videos with no delay.

## Results

### Sampling and molecular identification of host species from faecal samples

The dogs found 17 scat samples. Based on genetic identification, nine samples were classified as golden jackals’, four as red foxes’, one as a domestic dog’s and three as of unknown origin. The samples of which the species was not clarified might have been overmarked scats, which the dogs then misidentified. Identification of the individual animal was only possible for three of the nine golden jackal faecal samples. Each of them came from a different individual, two from females and one from a male (Table 1).

**Table 1.** Results of parasitological and molecular examination of golden jackal faecal samples from southern Poland

Sample I.D.	Individual	Coproscopy results (eggs per g of sample)				PCR identification*
		*Trichuris* sp.	Ancylostomatidae	*Toxocara* sp.	Capillariidae	
22_GS_18	n.a.	2.3	0.0	0.0	0.0	-
22_GS_19	n.a.	0.0	0.8	0.4	2.4	*Mesocestoides litteratus*
22_GS_21	PLG001f	0.0	0.0	0.0	1.9	-
22_GS_22	n.a.	0.0	0.0	0.0	0.0	*Mesocestoides litteratus*
22_GS_24	n.a.	0.0	0.5	0.0	0.0	-
22_GS_26	PLG002m	0.3	3.7	0.0	1.6	*Mesocestoides litteratus*
22_GS_31	n.a.	0.0	0.0	0.0	0.0	n/a
22_GS_35	PLG004f	0.0	8.6	0.0	0.0	n/a
22_GS_37	n.a.	0.0	0.0	0.0	46.3	n/a
Hunted jackal	n.a.	0.0	0.0	0.0	2.0	-
Hunted jackal	n.a.	0.0	15.0	0.0	0.0	*Taenia serialis*
		Range 0.3–2.3	Range 0.5–15.0	Range 0.0–0.4	Range 1.6–46.3	
		Prevalence 18.2%	Prevalence 45.5%	Prevalence 9.0%	Prevalence 45.5%	

1 * – qPCR for *Echinococcus multilocularis* (28), qPCR for *E. granulosus s.l*. (34) and multiplex PCR for *E. multilocularis, E. granulosus* and other tapeworms (48), completed by sequencing of obtained amplicons; n.a. – lack of sample for molecular study

## Parasitological examination of faecal samples

**Fig. 2.**
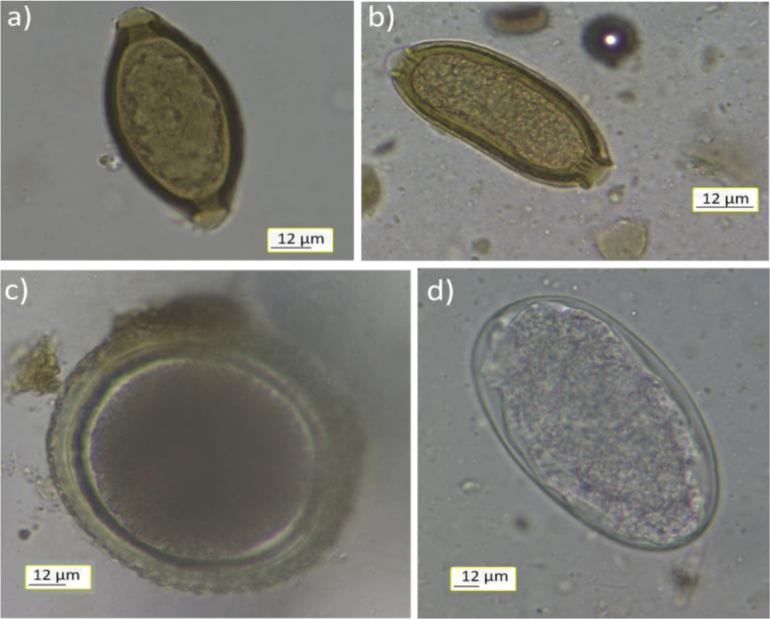
Parasite eggs found in Polish golden jackal faeces: a) *Trichuris* sp.; b) Capillariidae family; c) *Toxocara* sp.; d) Ancylostomatidae family. Magnification 400×

The eggs of gastrointestinal nematodes were found in 9 of the 11 faecal samples (81.8%) from golden jackals (Table 1). Coproscopic examination of the samples revealed the presence of eggs of *Toxocara* and *Trichuris* spp., and eggs attributable to the of Capillariidae and Ancylostomatidae families (Fig. 2, Table 1). Most prevalently found were eggs of Capillariidae and Ancylostomatidae nematodes (45.5%), whereas eggs of *Toxocara* spp. were the rarest ones (9.0%) (Table 1).

## Parasitological examination of the intestines

**Fig. 3.**
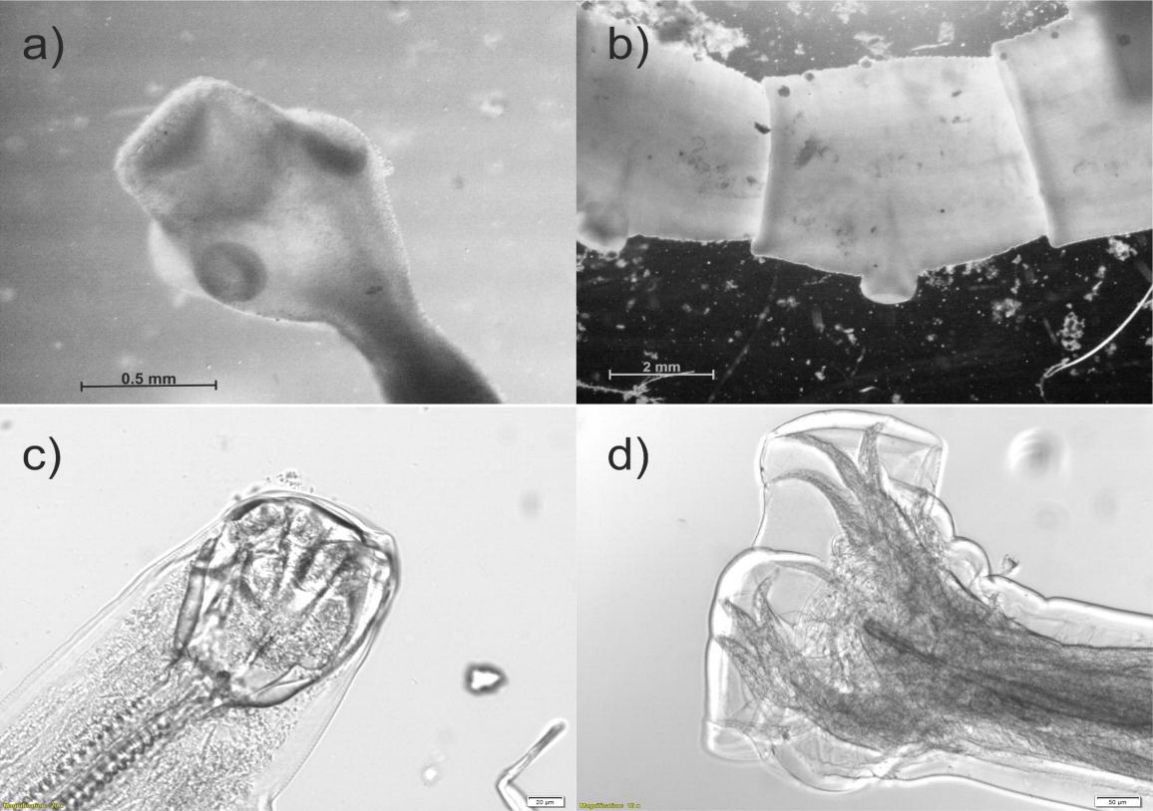
Helminths found in the intestine of a Polish golden jackal: a) *Taenia serialis* scolex and b) strobila, c) male *Uncinaria* sp. anterior extremity and d) posterior extremity

Examination by SCT of one of the individuals revealed the presence of one *Uncinaria* sp. nematode and six *Taenia serialis* tapeworms (Fig. 3). The species of *T. serialis* was confirmed by multiplex PCR (48) and sequencing. The intestines of the second individual were parasite free.

## Molecular screening for tapeworm presence

Molecular examination of faeces (using multiplex PCR and qPCRs) showed that all samples were negative for *Echinococcus* spp. However, the multiplex PCR followed by sequencing and comparison of the sequences with those in the GenBank database identified DNA of *Mesocestoides litteratus* in three faeces samples and *Taenia serialis* in the sample collected from the rectum of one hunted jackal with *T. serialis* tapeworms in its intestines (Table 1).

## Camera trapping

The camera traps recorded 2,105 videos of at least 11 carnivore species (including domestic cats and dogs). The most commonly recorded were the red fox (800 videos), golden jackal (612 videos), and marten (*Martes* sp. (species unresolved), 298 videos). Other carnivore wildlife included the European badger, Eurasian otter, raccoon dog, stoat (*Mustela erminea*), European polecat (*Mustela putorius*) and raccoon (*Procyon lotor*). Domestic cats were recorded 193 times at 21 locations, of which 19 were also visited by golden jackals during the monitoring period. Domestic dogs were captured on video 9 times at six sites, all of which were also golden jackal occurrence sites (Fig. 1).

In 2023, camera trap material confirmed the reproduction of at least two golden jackal family groups.

## Discussion

Our findings provide the first results on the parasite fauna of a new mammalian species in Poland, the golden jackal. These animals are still very rare in this part of Europe, and their presence is sometimes difficult to detect because of their elusive lifestyle (9, 12). This, combined with difficulties in distinguishing their scats from those of red foxes, renders it challenging to conduct faeces-based research. We used the most reliable methods for scat discovery and recognition of the origin animal species, which still resulted in a small sample size. However, despite the small sample size (two family groups and at least three individuals, making 11 samples), we believe the results, as the first from this new golden jackal habitat, provide significant insight into understanding the zoonotic potential of the parasites which the golden jackal carries.

We found four genera of gastrointestinal nematodes in golden jackal faeces. All of the identified parasites had been reported before in southern Poland and are widespread in wild carnivores (including the wolf, red fox and marten) (5). For example, the parasitic fauna of Polish red foxes, which have the highest dietary overlap with golden jackals (11, 33), are nearly identical to the parasites found in the examined faeces of the jackals (24).

We found Capillariidae eggs both in faecal samples from the environment and in the sample derived from the intestine of the hunted individual. This may suggest that the detected Capillariidae eggs were of *Eucoleus aerophilus* lungworms, because these nematodes are very common in red foxes in Poland and in the region close to our study area (62% prevalence) (41).

Special attention should be directed towards the presence of parasites with a zoonotic potential, *i.e. Toxocara* sp. and hookworms, in the faeces of golden jackals; however, it should be emphasised that foxes were the species most frequently recorded by camera traps and therefore represent an important species to consider when assessing these pathogen transmission chains. We found that the area used by the golden jackals was frequented by domestic cats, and to a lesser degree, dogs. Most free-ranging cats in Poland have a home, but in rural areas, they often move quite far away from their households (50). The dogs recorded in this study were either accompanied by people or were of hunting breeds, and therefore also had an owner. Thus, while domestic animals may act as epidemiological intermediates, the hypothesis cannot be evaluated based on the present data.

The low observed number of parasitic eggs in the faecal samples can be ascribed to several factors. The freezing and transportation of stool samples may have destroyed certain eggs. Moreover, the collection of material might have coincided with a phase in the parasite’s life cycle wherein egg laying did not occur, thereby precluding their detection in the faecal matter. Additionally, the microscopic inspection of faecal samples introduced the potential for laboratory errors as failures to detect eggs or incomplete identification of all egg species. The non-detection of *Mesocestoides* sp. and *Taenia* sp. eggs in faeces despite positive PCR results is also a result of the limitations of the flotation method, which is particularly unreliable for these cestode eggs. A similar methodological inferiority was observed in studies of foxes, where more than 20-fold fewer *Mesocestoides*-positive and 4-fold fewer *Taenia*-positive samples were found in flotation than in SCT (24).

We did not confirm the presence of *Echinococcus* spp. in samples from golden jackals. It must be stressed that the study area is characterised by a low (although increasing) prevalence of *E. multilocularis* also in the main definitive host, the red fox (25). Therefore, the occurrence of these tapeworms in the additional definitive host (jackal) is probably even rarer and would require testing a larger number of samples to prove. However, literature data indicate that jackals frequently host these dangerous parasites in Europe. In Switzerland in recent years, *E. multilocularis* was found in two out of five jackals (15), and in Hungary *E. multilocularis* was detected in 15.6% and *E. granulosus s.l*. in 1.7% % of golden jackals (3). This indicates the need for further epidemiological investigations, particularly since the population of jackals in Poland is likely to increase. An example of a species that has significantly increased its numbers and range of occurrence in recent decades in Poland and has transpired to be significantly infected with *E. multilocularis* and *E. granulosus s.l*. tapeworms is the grey wolf (26). This indicates that the risk of cross-species transmission and zoonotic acquisition of echinococcosis should not be ignored from the golden jackal host either.

## Conclusion

Our findings provide no evidence that golden jackals currently pose a novel threat to the health of wildlife or humans in Poland. Nevertheless, ongoing surveillance of diseases in this expanding species remains essential, particularly as golden jackals continue to colonise new areas, and may circulate parasites within an environment shared with domestic animals.
